# The Bristol Royal Infirmary

**Published:** 1888-01-21

**Authors:** Fairfax Goodall


### THE EDITOR'S LETTER-BOX.

### THE BRISTOL ROYAL INFIRMARY.

Some of your readers will, I am sure, be glad to learn that the commission for the Lysaght memorial window has been entrusted to the well-known firm of glass-painters, Messrs. Bell and Son, College Green, in this city. We confidently hope that the window will be placed in the chapel before Easter Day. It consists of three lights with tracery. The principal subject represents our Lord healing the sick, and is illustrative of St. Luke iv. 40: "Now when the sun was setting, all they that had any sick with divers diseases brought them unto Him; and He laid His hands on every one of them, and healed them." In smaller panels below are three subjects of Mercy. The central one, St. Peter and St. John healing the lame man at the Beautiful Gate of the Temple (Acts iii. 1-10), and on either side a scene from the parable of the Good Samaritan; first, the Good Samaritan succouring the wounded man (St. Luke x. 34); and next, the Good Samaritan paying the host (St. Luke x. 35). These are all set in panels formed by canopy work. In the tracery are emblems of Faith (cross), Hope (anchor), and Love (pelican feeding her young). The accessory ornament is of foliated design. I have received about eighty pounds towards the cost, which will amount to one hundred.—Very faithfully yours, Fairfax Goodall.

